# Technological Addictions: The New Frontier in Addiction Psychiatry

**DOI:** 10.1192/j.eurpsy.2024.845

**Published:** 2024-08-27

**Authors:** P. Levounis

**Affiliations:** Psychiatry, Rutgers New Jersey Medical School, Newark, United States

## Abstract

**Introduction:**

Addiction to video games, cybersex, internet gambling, social media, texting and emailing, and online auctions can be as addictive as substances. These technological addictions have real-world ramifications and lead to the loss of jobs, money, and loved ones. As technology becomes integrated into many facets of modern life, the appreciation of such addictions has become increasingly challenging. This session will explore the addictive potential of technology and discuss the legitimacy of technological addictions as psychiatric conditions worthy of medical assessment, diagnosis, and treatment.

**Objectives:**

1. List five forms of Technological Addictions as they appear in the scientific literature of 2023.
2. Describe the psychology and culture surrounding Internet Gaming addiction.
3. Distinguish between normal use and addiction.

**Methods:**

Lecture and discussion

**Results:**

1. Research on the phenomenology and nosology of these illnesses helps us further elucidate the distinction between problematic and nonproblematic use of technology, especially in children and young adults.
2. Another area of new research involves emerging technologies. By the time clinicians get a firmer grasp of today’s ailments, the technology of tomorrow—such as virtual reality and smart devices powered by artificial intelligence—will be commonplace enough to bring about a host of new problems.

**Conclusions:**

Though data on the prevalence of technological addictions are sparse, most people use computers, tablets, and smartphones regularly with great benefits and no serious adverse consequences. We will need to be ready to guide our patients, our colleagues, and the general public on how to best handle technology with an eye on maximizing its enormous potential for fulfillment, gratification, and happiness while minimizing its significant risks for dissatisfaction, misery, and despair.

**Disclosure of Interest:**

None Declared

